# TransmiR v2.0: an updated transcription factor-microRNA regulation database

**DOI:** 10.1093/nar/gky1023

**Published:** 2018-10-29

**Authors:** Zhan Tong, Qinghua Cui, Juan Wang, Yuan Zhou

**Affiliations:** Department of Biomedical Informatics, School of Basic Medical Sciences, Peking University, Beijing 100191, China

## Abstract

MicroRNAs (miRNAs) are important post-transcriptional regulators of gene expression and play vital roles in various biological processes. It has been reported that aberrant regulation of miRNAs was associated with the development and progression of various diseases, but the underlying mechanisms are not fully deciphered. Here, we described our updated TransmiR v2.0 database for more comprehensive information about transcription factor (TF)-miRNA regulations. 3730 TF–miRNA regulations among 19 species from 1349 reports were manually curated by surveying >8000 publications, and more than 1.7 million tissue-specific TF–miRNA regulations were further incorporated based on ChIP-seq data. Besides, we constructed a ‘Predict’ module to query the predicted TF–miRNA regulations in human based on binding motifs of TFs. To facilitate the community, we provided a ‘Network’ module to visualize TF–miRNA regulations for each TF and miRNA, or for a specific disease. An ‘Enrichment analysis’ module was also included to predict TFs that are likely to regulate a miRNA list of interest. In conclusion, with improved data coverage and webserver functionalities, TransmiR v2.0 would be a useful resource for investigating the regulation of miRNAs. TransmiR v2.0 is freely accessible at http://www.cuilab.cn/transmir.

## INTRODUCTION

MicroRNAs (miRNAs) are one class of endogenous short non-coding RNAs (∼22 nt) that typically mediate target mRNA degradation or translation inhibition by binding to the 3′-untranslated regions (3′-UTRs) (1). As important regulators of gene expression, miRNAs play crucial roles in a variety of biological processes, such as development, cell proliferation, differentiation, apoptosis and cellular signaling (2,3). In recent years, abundant evidence has shown that the deregulation of specific miRNA is associated with various diseases like cancers (2,4), cardiovascular diseases (5,6), and psychiatric disorders (7,8). However, the underlying mechanism of miRNA deregulation often remains elusive.

Comprehensive transcriptional networks of regulation between transcription factors (TFs) and miRNAs are necessary for understanding the deregulation of gene expression in different physiological and disease conditions (9–12). Although there exist abundant resources for miRNA target and transcriptomic profiling data, the knowledgebase for TF–miRNA regulations is far from sufficient. The reasons are two-folded. On the one hand, there are difficulties to gain accurate locations of miRNA gene transcription start sites (TSSs) and therefore the locations of miRNA promoters, because the 5′ end of primary miRNA transcript is often rapidly sliced by Drosha in the nucleus (13). Indeed, substantial efforts have been made to decipher TF–miRNA regulations by algorithms that combine ChIP-seq data, TF binding motifs and transcriptome profiles (14–18). For example, ChIPBase (15) provides regulatory relationships between TFs and non-coding RNAs by parsing ChIP-seq data. CircuitDB (19) predicted transcriptional regulatory circuits for human and mouse based on motif scanning analysis. Nonetheless, without accurate miRNA TSSs, it is hard to tell whether the parsed TF–miRNA regulations are truly functional. Thanks to the recent development of high-throughput deepCAGE sequencing technique, genome-wide accurate identification of miRNA TSSs has become possible (20). As the result, DIANA-miRGen (17) and mirTrans (16) were recently established to incorporate comprehensive knowledge about cell-specific miRNA TSSs and TF–miRNA regulations for human and/or mouse.

On the other hand, however, literature-derived TF–miRNA regulation data remain limited. Such data are critical for species where the genome-wide miRNA TSS data are not available (i.e. actually, all species other than human and mouse). In 2010, we established TransmiR v1.0 (21), one of the first TF–miRNA regulation databases, based on manual literature curation. Subsequently, several rounds of updates have been performed to the database. Here, we present the v2.0 version of TransmiR. With extensive manual curation, the literature-derived TF–miRNA regulation data from TransmiR v2.0 show 15-fold increase in comparison with TransmiR v1.0. Besides, by combining experimentally supported (for human, mouse and *Caenorhabditis elegans*) and predicted miRNA TSSs with the comprehensive ChIP-seq datasets, >1 million putative TF–miRNA regulations are further included for five species. The TF–miRNA regulations derived from ChIP-seq data were classified into level 1 (predicted) and level 2 (supported by high-throughput experimental data) according to the reliability of TSS annotations. Finally, multiple new functional modules like network visualization, TF–miRNA regulation prediction and enrichment analysis for TF regulations are provided in the updated web interface of the database, which will be described in the following sections.

## DATABASE CONSTRUCTION

### Literature-curated TF–miRNA regulation data

When building TransmiR v2.0, we started our manual curation procedure on the basis of TransmiR v1.2, which was launched in 2013. Therefore, we first searched PubMed records during 2013–2017 with the keyword ‘Transcription factor AND microRNA’ and retrieved >8000 records. Then, we manually surveyed all of these papers and curated TF–miRNA regulations that were supported by promoter-related experiments (e.g. ChIP and luciferase report assays detecting the activity of promoters). For each literature-curated TF–miRNA regulation record, we included the TF–miRNA regulation pair, type of regulation (i.e. activation or repression), PubMed ID and species information. Since TFs regulate miRNAs at the transcriptional level, we tried to map the TF–miRNA regulation annotations to the miRNA genes. In some special cases, one miRNA could be encoded by multiple miRNA genes, but the original publication did not specify the miRNA gene from which the miRNA was derived. We kept the literature-reported miRNA names for such special cases. Besides, the names of TFs were also standardized as official gene symbols. Furthermore, we incorporated external annotations, such as their functions and related diseases, for both the TFs and the miRNAs. The TFs were annotated with the following four items: Entrez gene ID, Ensembl gene ID, gene associated diseases (from DisGeNET database (22)), and cancer prognostic association data (from Human Protein Atlas database (23)). For TFs in non-human species, we tried to map the annotations according to their human orthologs recorded in OMA database (24). Each miRNA annotation entry includes the following four external items: miRBase ID, genome context of this miRNA gene, miRNA associated diseases from HMDD v2.0 database (25) and a link to miRBase database. Finally, experimentally validated miRNA-TF feedback regulations derived from miRNA target databases (TarBase (26) and miRTarBase (27)) were also included in TransmiR v2.0 for more comprehensive annotations.

### ChIP data-derived TF–miRNA regulations

According to the reliability of TSS annotations, the TF–miRNA regulations derived from ChIP-seq data were classified into level 1 (predicted) and level 2 (supported by high-throughput experimental data) regulations. For the level 1 records, we first downloaded all miRNA genome coordinate information from miRBase v22 (28) for five species, including *Homo sapiens, Mus musculus, Rattus norvegicus, Drosophila melanogaster* and *C. elegans*. A group of miRNAs that are located within 1kb of distance on the same genomic strand were defined as a miRNA cluster. For intergenic miRNAs, we chose 5′-end of the pre-miRNA or that of the first member in the miRNA cluster as the putative TSS. For intragenic miRNAs, we retrieved miRNA host genes from UCSC RefGene dataset (29), and 5′-end of the host gene was considered as the putative TSS. Next, a window from 5 kb upstream to 1 kb downstream of the miRNA TSS was identified as the putative promoter based on previous studies (18,30). Apparently, this definition could cover most of miRNAs, but suffered from substantial false positives. Therefore, for the level 2 records, the miRNA TSSs which were supported by high-throughput experiments (31,32) were firstly identified. And the 300 bp upstream to 100 bp downstream of each miRNA TSS was identified as the putative promoter (33). Due to the limitation of current high throughput data, the level 2 regulation records are only available for human, mouse and *C. elegans*. Finally, all promoter annotations were converted to the latest genome assemblies using UCSC liftOver tool, and regions that failed to be transferred were discarded.

To decode TF–miRNA regulations from ChIP-seq data, we first downloaded tissue-specific ChIP-seq data from the ChIP-Atlas database (http://chip-atlas.org/) with significance score threshold of 200 for the aforementioned five species. The ChIP-Atlas database has shown excellent coverage and acceptable accuracy in a recent evaluation (34). Genome coordinates of the ChIP-seq peaks were converted to be consistent with miRBase v22 with UCSC liftOver tool. BEDTools (35) was used to find the overlaps between ChIP-seq peak regions of TF binding and the miRNA promoters, and finally identify the putative TF–miRNA regulations from high-throughput data.

### Predicting TF–miRNA regulations based on TF binding motifs

To gain a more comprehensive information of TF–miRNA regulations in human, predicted TF–miRNA regulations based on TF binding motifs were also provided. We first downloaded the highly conservative TF binding motif data from UCSC genome browser and scanned the miRNA promoters in human genome to obtain the predicted TF–miRNA regulations. The raw score and the normalized Z-score of the predicted TF binding site were obtained according to previous protocol (36). The predicted TF–miRNA regulation data were classified and annotated based on the pipeline described above.

### Construction of disease-specific TF–miRNA regulatory networks

Based on experimentally supported TF-disease associations and miRNA-disease associations, we tried to construct disease-specific TF–miRNA regulatory networks. Because diseases recorded in HMDD v2.0 and DisGeNET database used different disease vocabularies, we first mapped the disease names to the MeSH terms. As the result, 45 diseases which had both disease-associated TF and disease-associated miRNA information were retained. For 24 of them, there were sufficient regulatory relationship records between the TFs and miRNAs in our database. Therefore, we constructed disease-specific TF–miRNA regulatory networks for these diseases.

### Collection of miRNA sets and enrichment analysis

MiRNA sets are defined as groups of miRNAs that have shared functional associations. Here, miRNA sets were assembled according to the regulating TFs. Based on the classification of ChIP-seq derived TF–miRNA regulation data, we provided two types of miRNA sets, i.e. ‘Set level 1′ and ‘Set level 2′. The ‘Set level 2′ are limited to literature-curated TF–miRNA regulations and the level 2 ChIP-seq derived TF–miRNA regulations described above, while the ‘Set level 1′ contains all TF–miRNA regulation data. Finally, hypergeometric test (37) was used to determine the overrepresented miRNA sets (i.e. the overrepresented regulating TFs) among a miRNA list of interest. And the *P*-values for all miRNA sets were adjusted by Bonferroni and FDR corrections, respectively.

### Database implementation

In TransmiR v2.0, all of the data tables were organized with SQLite, a lightweight database management system. The website was developed based on Django, a Python-originated framework. D3.JS was used to visualize the TF–miRNA regulatory networks. The database is available at http://www.cuilab.cn/transmir.

## DATABASE OVERVIEW AND USAGE

### Overview of TransmiR v2.0 database

**Figure 1.**
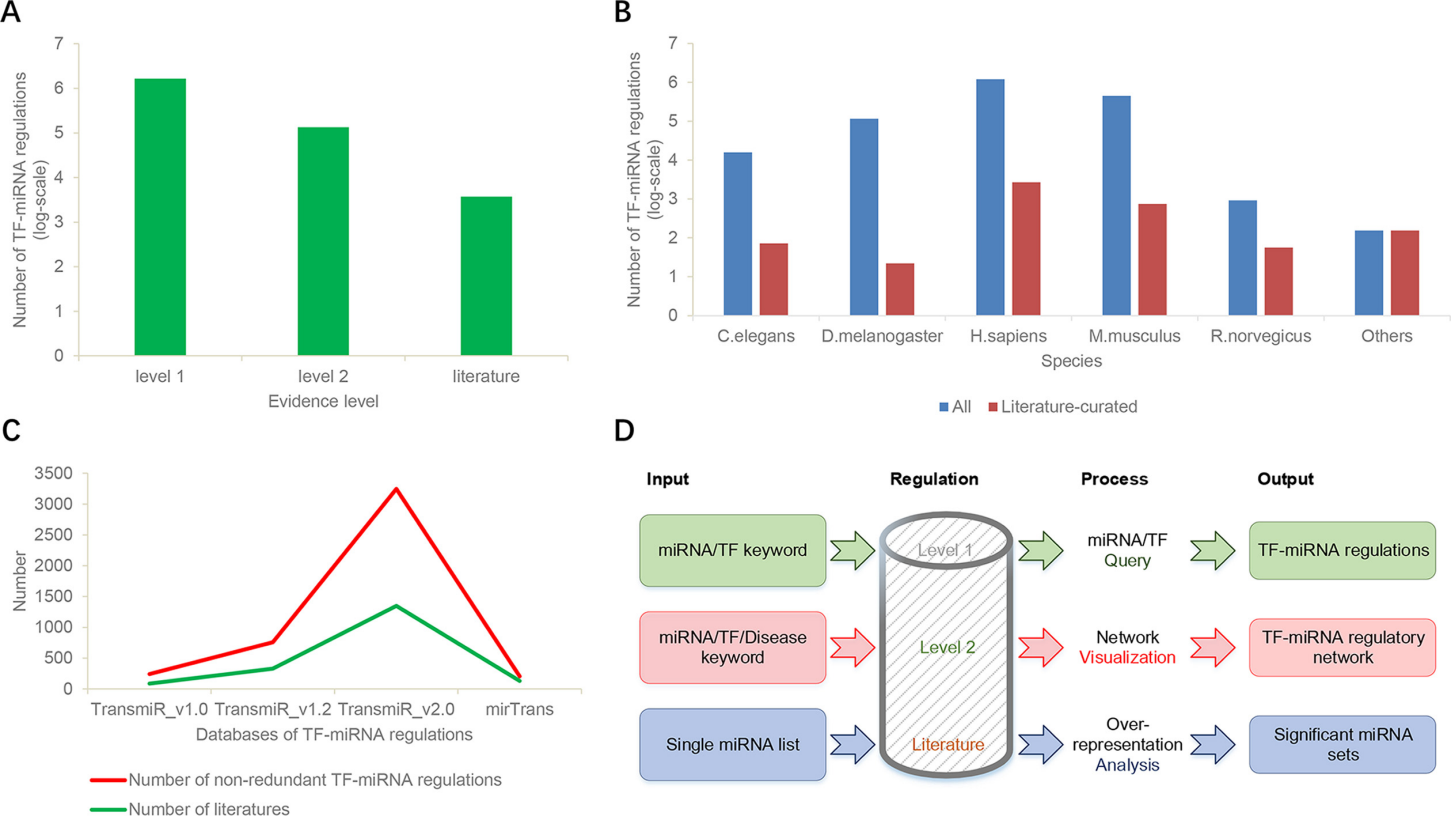
The overview of TransmiR v2.0 database. (**A**) The composition of transcriptional regulation data in TransmiR v2.0 database. (**B**) The distribution of literature-curated TF–miRNA regulation data and all evidence level TF-miRNA regulation data among different species. (**C**) The comparison of non-redundant numbers of the literature-curated TF–miRNA regulations and the related publications, across different databases. (**D**) The workflow of TransmiR v2.0 database. TransmiR v2.0 database website provides three major functionalities: (i) database query; (ii) network visualization and (iii) enrichment analysis.

In this major update to TransmiR v2.0, we manually curated 2852 TF–miRNA regulations from 1045 publications during 2013–2017 and included ChIP-seq derived TF–miRNA regulation records. Currently, TransmiR v2.0 contains 3730 literature-curated TF–miRNA regulations, covering 623 TFs, 785 miRNAs, 19 organisms and 1349 publications. Besides, we also provide >1.7 million TF–miRNA regulations derived from ChIP-seq evidence in five species (*H. sapiens, M. musculus, R. norvegicus, D. melanogaster* and *C. elegans*). Based on the reliability of the miRNA promoter annotations used, we further classified the TF–miRNA regulations derived from ChIP-seq data into level 1 and level 2 regulations (the level 2 promoter is supported by high-throughput experimental data, see more details in the 'Database Construction' section). The distribution of TF–miRNA regulation data at different levels of evidence is shown in Figure 1A. The low confidence, level 1 regulation records are more than twelve times as many as the higher confidence, level 2 records (1 651 502 versus 134 496). The literature-curated TF–miRNA regulations are orders of magnitude less than the ChIP-seq derived data for all species (Figure 1B). Nevertheless, when compared to the previous TransmiR v1.0, TransmiR v1.2 and mirTrans databases, the updated TransmiR v2.0 provides the most abundant resource for literature-curated TF–miRNA regulations (Figure 1C), with >10-fold growth of non-redundant TF–miRNA regulations compared to TransmiR v1.0. The quality of TF–miRNA annotations is also improved in comparison with the previous versions. First, heterogeneous TF and miRNA names from literatures are standardized as the gene official symbols in NCBI and the miRNA names in miRBase v22, respectively. Second, external annotations such as miRNA/gene–disease associations, cancer prognostic associations and miRNA-TF feedback regulation data are included. Third, for the ChIP-seq derived TF–miRNA regulations and the predicted TF–miRNA regulations based on binding motif matrices of human TFs, the genome locations and sequences of TF binding sites are also provided.

### Database usage

**Figure 2.**
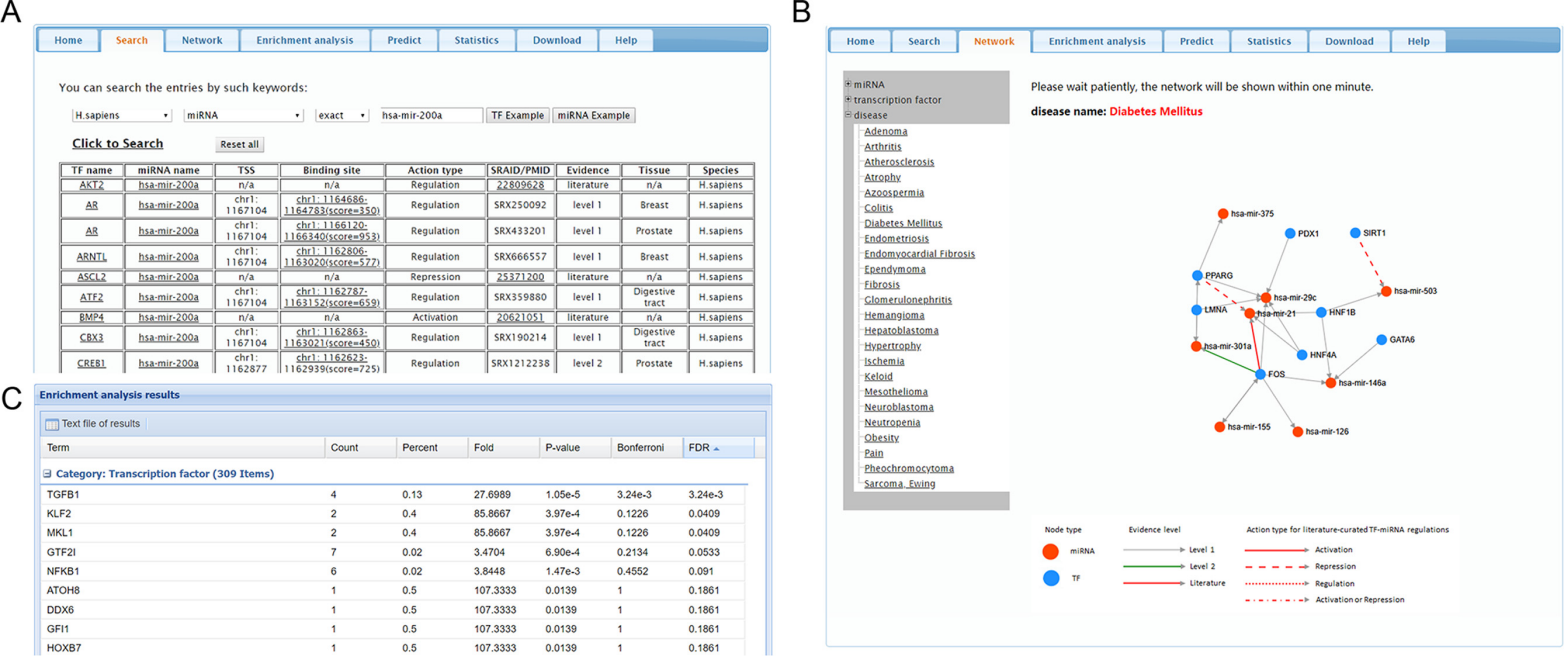
The sample results of TransmiR v2.0 database. (**A**) The sample database query (by TF or miRNA) result. (**B**) The sample network view of TF–miRNA regulations. (**C**) The sample tabular view of enrichment analysis result (sorted by FDR).

The workflow of TransmiR v2.0 is shown in Figure 1D. Depending on the input, TransmiR v2.0 provides three major functionalities: (i) database query, (ii) network visualization and (iii) enrichment analysis. First, if users input single miRNA or TF name, we provide a ‘fuzzy search’ function to search the database by the full or partial names of TFs or miRNAs in the ‘Search’ page. Each entry of search result contains nine items, which are TF official symbol, miRNA name, TSS location, TF binding site, action type (activating, repressing or non-specified regulation, with/without feedback regulation), SRAID/PMID, evidence, tissue and species (Figure 2A). The search result can also be downloaded by clicking the hyperlink below the entry table. The predicted TF–miRNA regulations can be queried in a similar way from the ‘Predict’ page. Each entry of the search result contains eight items, which are TF official symbol, miRNA name, TSS location, TF binding site, action type (non-specified regulation only, with/without feedback regulation), evidence, raw score and normalized *Z*-score. For the results from both the ‘Search’ page and the ‘Predict’ page, the TF binding site sequences can be obtained through the hyperlinks on the genome locations of TF binding sites. Second, the TF–miRNA regulation data related one TF, one miRNA or one disease can also be graphically visualized in the ‘Network’ page (Figure 2B). In disease-specific TF–miRNA regulatory networks, all regulations between the TFs and the miRNAs that are related to the same disease are depicted. One may intuitively infer key regulators and regulatory interactions from such disease-specific TF–miRNA regulatory networks. For example, in the TF–miRNA regulatory network of diabetes mellitus, we found all of the TFs (FOS, PPARG, HNF1B) with high degree centralities were validated to be associated with diabetes mellitus in related publications (38–40). Abundant evidence has suggested that hsa-mir-21 was up-regulated in diabetic patients (41). According to the TransmiR v2.0 record, it could be transcriptionally activated by c-Fos. Interestingly, previous reports suggested that insulin stimulation could markedly increase c-Fos messenger RNA level in adipose and muscle tissue of diabetic animals (38,42). Therefore, one could infer that the activation of hsa-mir-21 may partly by the increased amount of c-Fos in diabetic patients. And this regulation may play an important role in insulin signaling. For example, the defects of PPAR-gamma were suggested to be associated with increased risk of type 2 diabetes (43), but the underlying mechanism was unclear. We noted that PPAR-gamma is capable to decrease the transcriptional activity of c-Fos (44). Therefore, one plausible mechanism would be that the defects of PPAR-gamma might cause the activation of c-Fos and then up-regulation of hsa-mir-21, which might finally result in increased diabetes risk. Third, if users input a list of miRNAs, an enrichment (overrepresentation) analysis of TF regulations can be performed via the ‘Enrichment analysis’ page. Users can browse the result of enrichment analysis in a new tabular view, where the miRNA set name (i.e. the regulating TF) and the associated statistics (*P*-value, Bonferroni and FDR) are shown (Figure 2C). To exemplify the usage, we manually collected the de-regulated miRNAs in hepatocellular carcinoma from the previous study (45), which were validated by qRT-PCR and The Cancer Genome Atlas (TCGA) dataset. Then, we kept ‘size of miRNA category’ setting as default and chose ‘Set level 1′ as the background sets to perform the enrichment analysis. We found all the significant TFs with FDR < 0.05 (i.e. TGFB1, KLF2, MKL1), were closely related to hepatocellular carcinoma (46–48), supporting the predictions from the enrichment analysis. Finally, the more detailed tutorial for the usage of TransmiR v2.0 is available in the ‘Help’ page of the website.

## CONCLUSION

Here, we present TransmiR v2.0 database. To our knowledge, TransmiR v2.0 provides the most abundant resource for literature-curated TF–miRNA regulation data, which covers a variety of species. TransmiR v2.0 database also includes the ChIP-seq derived TF–miRNA regulations and the predicted TF–miRNA regulations based on the binding motif matrices of human TFs, which greatly expands our knowledge of TF–miRNA regulations. Furthermore, TransmiR v2.0 provides more additional functionalities, ‘Network visualization’ and ‘Enrichment analysis’, which enable better applications of our datasets for hypothesis generation and result interpretation. For example, users may infer potential key regulators and regulatory relationships in the disease-specific TF–miRNA regulatory networks. And if users have a list of de-regulated miRNAs in one specific disease condition, the potential disease-related regulating TFs could be identified by the enrichment analysis. Finally, all the data in TransmiR v2.0 database are freely available for academic usage. Users can download the datasets for further analysis.

However, there are still some limitations in TransmiR v2.0 database. First, our definition of miRNA gene TSSs suffers from inaccuracy of high-throughput data. More importantly, the experimentally supported miRNA gene TSSs data are still limited and not always tissue-specific. Some other related databases may complement this weak point. For example, mirTrans includes computational annotations of cell-specific miRNA gene TSSs based on H3K4me3 and DHS data. And DIANA-miRGen v3.0 provides accurate cell-line-specific miRNA gene TSSs based on RNA-seq, ChIP-seq and DNase-seq datasets. Second, the ChIP-seq derived TF–miRNA regulations and the predicted TF–miRNA regulations based on the binding motif matrices of human TFs do not specify the action type (i.e. activation or repression). One alternative solution for this problem is to integrate our TF–miRNA regulation data with specific transcriptome dataset to compute the expression correlations between the TF and miRNA pairs. Third, although the PubMed IDs and regulatory types are supplied for our literature-curated TF–miRNA regulations, there is no benchmarking standard to evaluate the confidence of the regulations. In the future, more detailed information of literature-curated TF–miRNA regulations will be included, which will make our manually surveyed regulation data more reliable and enable better applications of this dataset for bioinformatics analysis.

With the development of experimental and computational methodology, we will continue to improve and expand our literature-curated dataset and regulatory datasets derived from ChIP-seq data. We believe that TransmiR v2.0 database will provide more helpful resources to the community as it integrates more high-quality datasets and more powerful tools in the future.
